# The Role of *Salmonella* Genomic Island 4 in Metal Tolerance of *Salmonella enterica* Serovar I 4,[5],12:i:- Pork Outbreak Isolate USDA15WA-1

**DOI:** 10.3390/genes11111291

**Published:** 2020-10-30

**Authors:** Bradley L. Bearson, Julian M. Trachsel, Daniel C. Shippy, Sathesh K. Sivasankaran, Brian J. Kerr, Crystal L. Loving, Brian W. Brunelle, Shelby M. Curry, Nicholas K. Gabler, Shawn M. D. Bearson

**Affiliations:** 1 USDA, ARS, National Laboratory for Agriculture and the Environment, Agroecosystems Management Research Unit, Ames, IA 50011, USA; brian.kerr@usda.gov (B.J.K.); shelby.curry@biomin.net (S.M.C.); 2 USDA, ARS, National Animal Disease Center, Food Safety and Enteric Pathogens, Ames, IA 50010, USA; julian.trachsel@usda.gov (J.M.T.); daniel.shippy@yahoo.com (D.C.S.); sathesh.sivasankaran@usda.gov (S.K.S.); crystal.loving@usda.gov (C.L.L.); brunelle@arborbiosci.com (B.W.B.); shawn.bearson@usda.gov (S.M.D.B.); 3 Genome Informatics Facility, Iowa State University, Ames, IA 50011, USA; 4 Animal Science Department, Iowa State University, Ames, IA 50011, USA; ngabler@iastate.edu

**Keywords:** *Salmonella*, metal tolerance, mobile genetic element, conjugation, copper

## Abstract

Multidrug-resistant (MDR; resistance to >3 antimicrobial classes) *Salmonella enterica* serovar I 4,[5],12:i:- strains were linked to a 2015 foodborne outbreak from pork. Strain USDA15WA-1, associated with the outbreak, harbors an MDR module and the metal tolerance element *Salmonella* Genomic Island 4 (SGI-4). Characterization of SGI-4 revealed that conjugational transfer of SGI-4 resulted in the mobile genetic element (MGE) replicating as a plasmid or integrating into the chromosome. Tolerance to copper, arsenic, and antimony compounds was increased in *Salmonella* strains containing SGI-4 compared to strains lacking the MGE. Following *Salmonella* exposure to copper, RNA-seq transcriptional analysis demonstrated significant differential expression of diverse genes and pathways, including induction of at least 38 metal tolerance genes (copper, arsenic, silver, and mercury). Evaluation of swine administered elevated concentrations of zinc oxide (2000 mg/kg) and copper sulfate (200 mg/kg) as an antimicrobial feed additive (Zn+Cu) in their diet for four weeks prior to and three weeks post-inoculation with serovar I 4,[5],12:i:- indicated that *Salmonella* shedding levels declined at a slower rate in pigs receiving in-feed Zn+Cu compared to control pigs (no Zn+Cu). The presence of metal tolerance genes in MDR *Salmonella* serovar I 4,[5],12:i:- may provide benefits for environmental survival or swine colonization in metal-containing settings.

## 1. Introduction

*Salmonella* is the most common bacterial causative agent of human foodborne illness, with an estimated 1.3 million *Salmonella* infections and 420 deaths annually in the US. [1]. Of the >2600 serovars of *Salmonella*, numerous serovars can cause human gastrointestinal disease, but many of these same serovars sub-clinically colonize food-producing animals [2,3]. This commensal-like colonization of food animals can result in unintentional contamination of subsequent food products at multiple stages along the food chain. The Center for Disease Control and Prevention’s National Outbreak Reporting System (NORS) reported 1798 *Salmonella* outbreaks from 2010 to 2017 [4], and several of those outbreaks were associated with the consumption of food animal products such as turkey, chicken, ground beef, shell eggs, and pork.

A multistate (WA, OR, ID, CA, AK) outbreak of multidrug-resistant (MDR) *S. enterica* serovar I 4,[5],12:i:- occurred in 2015 due to the consumption of contaminated pork, resulting in 188 human infections, 30 hospitalizations, and the recall of 523,380 lbs. of meat [5]. The MDR serovar I 4,[5],12:i:- isolates involved in the outbreak encoded *bla*_TEM−1_, *strAB*, *sul2*, and *tet*(B) genes for resistance to ampicillin, streptomycin, sulfisoxazole, and tetracycline (R-type ASSuT). Like other serovar I 4,[5],12:i:- strains, the outbreak isolates lacked the phase 2 flagellin FljB, and were therefore monophasic variants of *Salmonella enterica* serovar Typhimurium (serovar Typhimurium) [6].

Over the last two decades, the prevalence of serovar I 4,[5],12:i:- isolates in food-producing animals, as well as the isolation frequency of this serovar from human illness associated with foodborne transmission, has increased globally [7,8]. Based on the National Antimicrobial Resistance Monitoring System (NARMS), serovar I 4,[5],12:i:- prevalence increased 3.1-fold between 2003 (2%) and 2015 (6.3%) in the United States [9], ranking it the fourth most common nontyphoidal *Salmonella* serovar following serovars Enteritidis (19.9%), Typhimurium (10.6%), and Newport (9.8%). NARMS also reports that the emergence of serovar I 4,[5],12:i:- corresponds with an 8-fold increase in multidrug resistance from 8.3% in 2004 to 67.8% in 2015 and accounted for serovar I 4,[5],12:i:- being the most frequent nontyphoidal MDR serovar in 2015. Approximately one-third (34.5%) of all MDR nontyphoidal *Salmonella* isolates in 2015 were serovar I 4,[5],12:i:-.

Recently, we reported the genome sequence of MDR serovar I 4,[5],12:i:- strain USDA15WA-1, which was isolated during a USDA, Food Safety and Inspection Service (FSIS) investigation at the swine slaughter facility associated with the 2015 pork outbreak [6]. Our genomic analysis indicated that strain USDA15WA-1 contains two genetic insertions in its chromosome that are of potential interest for characterization. The first insertion is a ~28 kb MDR module inserted in the *fljB* region that deletes a portion of the ancestral genome (based on homology to serovar Typhimurium strain LT2) including the *fljB* gene; the monophasic phenotype of the pork outbreak-associated strain USDA15WA-1 is due to this genetic event. The MDR module encodes antimicrobial resistance for R-type ASSuT as well as tolerance genes for the metal mercury. An MDR module containing the same repertoire of genes for antimicrobial resistance and mercury tolerance has been described in a German serovar I 4,[5],12:i:- strain, 07-02006, isolated from a swine lymph node [10]. The second genetic insertion in strain USDA15WA-1 is *Salmonella* Genomic Island 4 (SGI-4), an ~80 kb mobile genetic element (MGE) that encodes genes for tolerance to the metals copper, silver, and arsenic. An investigation of MDR serovar I 4,[5],12:i:- strains isolated in the United Kingdom and Italy described the presence of SGI-4 in numerous isolates including strain SO4698-09 [11], and an investigation from Japan has also reported the presence of the MDR module and SGI-4 in serovar I 4,[5],12:i:- strains [12]. The presence of both SGI-4 and the MDR module in strain USDA15WA-1 suggests that this pork outbreak-associated isolate is related to globally distributed serovar I 4,[5],12:i:- strains. Multiple investigations indicate that a global increase in the use of elevated concentrations of zinc and copper as antimicrobials in the diets of food-producing animals may select for the acquisition of metal tolerance genes like those encoded in SGI-4 and the MDR module of strain USDA15WA-1 [11,13,14].

In the current study, we investigated the conjugative ability of SGI-4, the role of SGI-4 in copper tolerance, the response of serovar I 4,[5],12:i:- to copper exposure, and the effect of elevated copper and zinc in the diet of weanling piglets on the gastrointestinal microbiota, as well as serovar I 4,[5],12:i:- colonization and fecal shedding. Our data indicate that the presence of SGI-4 enhances metal tolerance in the Salmonella serovar I 4,[5],12:i:- isolate USDA15WA-1; acquisition of enhanced metal tolerance may have provided this serotype with a competitive advantage in production environments where metals are used as an antimicrobial agent, thereby offering a possible explanation for the increase in prevalence of this *S.* serovar in swine.

## 2. Materials and Methods

### 2.1. Bacterial Strains, Mutant Construction, and Primers

*S.* strains of MDR serovar I 4,[5],12:i:-, serovar Typhimurium, or their derivatives are shown in Table 1. Bacterial strain construction was performed using recombineering or conjugation. Primers for the polymerase chain reaction (PCR) amplification of a previously described universal knockout fragment (oBBI 92/93-neo) are indicated in Table S1 [15]. Gene deletion or targeted insertion of the neo gene conferring kanamycin resistance was performed as previously described [15] using pKD46-Gm [16].

### 2.2. Conjugation Studies

A pork outbreak-associated MDR serovar I 4,[5],12:i:- USDA15WA-1 donor strain marked with kanamycin resistance (BBS 1356) and nalidixic acid-resistant *Salmonella* Typhimurium UK-1 recipient strain (BSX 120) lacking the SGI-4 metal tolerance island were grown in Luria-Bertani (LB) broth to O.D._600_ = 0.4 (mid-log phase growth). All incubations were performed at 37 °C. Equal volumes of donor and recipient strains were aliquoted and mixed in a microfuge tube and 10 μL of the mixture was spotted on a Petri plate containing LB agar medium. Donor and recipient controls were also spotted individually to LB agar medium. Following overnight incubation, cell growth from the conjugation and control plates was scraped and individually suspended in phosphate buffered saline (PBS) buffer. The conjugation mixture and controls were plated on LB agar medium containing both kanamycin and nalidixic acid, ampicillin alone (for selection of the MDR module), or nalidixic acid alone to select for transconjugates, donors, or recipients, respectively; cultures were incubated overnight. The conjugation frequency was determined by dividing the colony forming units (CFU)/mL of transconjugates by the CFU/mL of donors for 7 replicates.

### 2.3. DNA Sequencing and Assembly

Genomes were sequenced to determine the location of SGI-4 in the transconjugates. Genomic DNA from *Salmonella* strains was extracted using the Roche High Pure PCR Template Preparation Kit (Roche Diagnostics GmbH, Mannheim, Germany) according to the manufacturer’s instructions following growth of an overnight culture in LB broth at 37 °C. Nucleotide sequences were generated on an Oxford Nanopore GridION X5 Sequencer (Oxford Nanopore Technologies, Oxford, UK) at the Iowa State University DNA Facility (Ames, IA, USA) and an Illumina MiSeq (San Diego, CA, USA) using a V3 reagent kit at the National Animal Disease Center Genomics Facility (Ames, IA, USA). Sequencing adapters and artifacts were removed from the short reads using BBtools [19]. Both long and short reads were used in a hybrid assembly with unicycler v 0.4 [20], which resulted in complete, closed genomes (and plasmids) (see Appendix A for Bioproject information). To confirm the multiplicity of plasmids in the assemblies, the short reads were mapped back to the final unicycler assemblies with BBMap and the average coverage of each plasmid was divided by the average coverage of the chromosome. To determine the location of SGI-4 in genomic and plasmid assemblies, a Blastn [21] search was employed, using the SGI-4 sequence from strain USDA15WA-1 (CP040686). Manual interrogation of the chromosomal region flanking SGI-4 was performed for strains BBS 1358^large^, BBS 1359, and BBS 1407 to identify the tRNA insertion site for the MGE.

### 2.4. Phenotypic Microarrays

To determine bacterial sensitivity or tolerance to specific chemical compounds, Phenotype Microarray assays (Biolog, Hayward, CA, USA) were performed using microplates PM9–PM20 with incubation and automated data collection in a Biolog GEN III OmniLog System. The concentration of chemical compounds in the microplates is proprietary but was chosen by the manufacturer to span a biologically relevant range over 4 wells. The microplates were inoculated as described by the manufacturer and incubated at 33 °C for 48 h with plate readings at 15 min intervals. Biolog phenotype microarray data were analyzed in R 3.6.0 [22] using the package “opm” [23]. Raw data were processed and normalized in accordance with package recommendations. Briefly, aggregated curve parameters were calculated with the “opm-fast” option. The normalized maximal growth curve value “A” was used to compare the growth phenotype between groups of strains. To assess statistical significance, a series of T-tests was performed comparing the maximal growth curve values for strains with SGI-4 to those without SGI-4 for each compound. Once testing was complete, *p*-values were corrected for multiple testing according to the false discovery rate (FDR) method. Compounds with FDR-corrected *p*-values less than 0.05 were considered significantly different between the groups.

### 2.5. Transcriptional Analysis of USDA15WA-1 in Response to Copper Exposure

USDA15WA-1 (SX 238) was streaked onto a solid LB agar plate (Invitrogen, Carlsbad, CA, USA), and an individual colony was selected for growth at 37 °C in LB broth overnight with agitation. Overnight cultures were diluted 1:200 in LB and grown to an OD_600_ of 0.15 (early log phase growth) at 37 °C with agitation. Copper II Sulfate solution (CuSO_4_) (Sigma, St. Louis, MO, USA) was added for final concentrations of 0 and 5 mM, and the cultures were incubated at 37 °C with agitation for 30 and 60 min. An aliquot of each culture was placed in RNA Protect (Qiagen, Germantown, MD, USA) and processed per the manufacturer’s instructions. Experiments were repeated three times (three biological replicates). RNA was extracted from bacterial pellets using the RNeasy mini kit (Qiagen, Germantown, MD, USA), followed by treatment with Turbo DNase (Ambion, Austin, TX, USA) to remove genomic DNA. A 2100 Bioanalyzer (Agilent technologies, Santa Clara, CA, USA) was used to evaluate total RNA. Bacterial ribosomal RNA (rRNA) sequences were depleted using the Ribo-Zero rRNA removal kit (Illumina, Inc., San Diego, CA, USA), and the quality of the rRNA removal step was assessed with the 2100 Bioanalyzer. Libraries were constructed using the NEB ultra II directional RNA library prep kit (Illumina, Inc., San Diego, CA, USA) and were sequenced at the Iowa State University DNA facility on an Illumina HiSeq 3000 (150 cycles, single-end reads). RNA-seq reads (see Appendix A for Bioproject information) were aligned to the reference genome of *Salmonella enterica* serovar I 4,[5],12:i:- strain USDA15WA-1 [6] using Segemehl [24] with default parameters. Read counts (number of reads that aligned to a specific gene) for each gene were quantified using in-house Perl scripts. Only uniquely mapped reads were used for downstream computational analysis. Read counts for each sample were normalized using the Bioconductor package DESeq2 v1.22.2 [25] run with R software v3.5.2. Principal component analysis (PCA) was performed to determine any expression outliers based on overall gene expression counts. Linear regression models within DESeq2 were used to identify differentially expressed genes between +/− CuSO_4_-exposed samples. Final values for differential expression were adjusted with a Benjamini–Hochberg (false discovery rate; FDR) with a *p*-value < 0.05 as significant. To identify functional consequences of the differentially expressed genes, a GO term [26,27] enrichment analysis was conducted. First, GO terms were assigned to genes in the USDA15WA-1 genome with interproscan [28]. Then, the R package topGO [29] was used to identify GO terms that were enriched in each group based on the differential expression data. This analysis was conducted for all three major GO aspects, “Biological processes”, “Molecular function”, and “Cellular Component”. Tests for enrichment were conducted using topGO’s “elimination” algorithm and the “Fisher” statistic.

### 2.6. Swine Study

One hundred crossbred pigs (equal barrows and gilts), 19–21 days of age, from a commercial farm were weaned and shipped to the Iowa State University Swine Nutrition Farm (Ames, IA, USA) and randomly distributed five pigs per pen. The basal swine diet included a trace mineral mix (Cu, Fe, I, Mn, and Zn) with 165 mg/kg Zn and 16.5 mg/kg Cu. Pigs in the control group (*n* = 10 pens) received the basal diet. The ration for the remaining pigs (*n* = 10 pens) was a modified basal diet with elevated concentrations of Zn and Cu (Zn+Cu). The Zn+Cu diet included an additional 2000 mg/kg ZnO and 200 mg/kg CuSO_4_ 5H_2_O with final metal concentrations of 1771 ppm Zn and 67.4 ppm Cu. Following four weeks of in-feed treatment, one pig from each of the 10 treatment pens receiving in-feed Zn+Cu and one pig from each of the 10 control pens were transported to the National Animal Disease Center, Ames, IA, and housed in isolation rooms based on treatment group (i.e., ten Zn+Cu pigs in one isolation room and ten control pigs in another isolation room). Feeding of the Zn+Cu and control diets continued for their respective groups for the remainder of the study. At ~7 weeks of age (4 weeks on treatment), all 20 pigs were inoculated via the intranasal route with 8 × 10^7^ CFU of the *Salmonella* I 4,[5],12:i:- strain SX 240 (swine passaged USDA15WA-1) [17]. Fecal sampling intervals for microbiota and *Salmonella* qualitative and quantitative bacteriology analyses included 0, 2, 7, 14, and 21 days post-inoculation (d.p.i.). At day 0 (prior to challenge), pigs tested fecal-negative for *Salmonella* as described previously [30]. At 21 d.p.i., all pigs were euthanized and necropsied to obtain tissue samples of the cecum, ileocecal lymph nodes (ICLN), Peyer’s patch region of the ileum, and contents of the cecum (1 g); all samples were evaluated by quantitative and qualitative bacteriology analyses as previously described [31] using XLT-4 medium (Becton, Dickinson and Co., Sparks, MD) supplemented with 50% tergitol, ampicillin (100 µg/mL), tetracycline (15 µg/mL), novobiocin (50 µg/mL), and streptomycin (50 µg/mL). Suspected *Salmonella* colonies were evaluated on BBL^™^ CHROMagar^™^ *Salmonella* (Becton, Dickinson and Co.) for mauve colonies indicative of *Salmonella*. All experimental procedures involving pigs were in compliance with the recommended principles described in the Guide for the Care and Use of Laboratory Animals by the National Research Council of the National Academies and were approved by the Institutional Animal Care and Use Committee at Iowa State University and the Animal Care and Use Committee at the USDA-ARS, National Animal Disease Center. Statistical analysis was performed using GraphPad Prism 5.01 (GraphPad Software, Inc., La Jolla, CA, USA). *p*-values less than 0.05 were considered significant. Statistical analysis of serovar I 4,[5],12:i:- quantitation in fecal, tissue, and cecal content samples was performed using an unpaired t-test. The shedding dynamics of *Salmonella* over time were investigated using a linear mixed model that allowed for interactions between treatment and day while including the individual animal as an error term. The R package lme4 [32] was used to fit this model using the function call lmer (log_sal ~ day * treatment + (1|pignum)). The lmerTest [33] package was used to assess the significance of the coefficients of the model.

### 2.7. Microbiota Analyses

16S rRNA gene amplicons of the V4 region were generated from feces, cecal contents, cecal mucosal, and ileal mucosal samples from the Zn+Cu and control groups (*n* = 10 pigs/group) as described in [34]. Briefly, samples were collected, immediately placed on ice, and stored at −80 °C until DNA extraction with Qiagen fecal microbiome kits. Amplicons were sequenced on an Illumina Miseq using V2 reagent kits (2 × 250 bp read lengths) (see Appendix A for Bioproject information). OTUs were generated with mothur [35] and classified using the SILVA v132 taxonomy. Global singletons were removed and samples with fewer than 2000 total reads were omitted.

The R package vegan [36] was used to assess the effect of treatment on community structure similarity through PERMANOVA tests on Bray–Curtis dissimilarities as implemented by the adonis function. Communities were rarefied to 2910 reads each and testing was performed. The overall effect of treatment and time was assessed with a global test followed by individual pairwise post hoc tests for comparisons of interest. *p*-values were corrected for multiple comparisons using the FDR method.

Differential abundance was accomplished using the DESeq2 package [25] using Wald tests with parametric fits and FDR-corrected *p*-values. Before testing, OTUs with fewer than 10 counts globally were removed and the resulting unrarefied counts were used as the input for DESeq2. The package phyloseq [37] was used to assist in data formatting.

## 3. Results and Discussion

### 3.1. Salmonella Genomic Island 4 Transferred by Conjugation from Strain USDA15WA-1 to Serovar Typhimurium

Genome sequencing and analysis of MDR *Salmonella* serovar I 4,[5],12:i:- strain USDA15WA-1 associated with the 2015 U.S. pork outbreak revealed the presence of SGI-4, a mobile genetic element containing multiple metal tolerance genes [6]. To determine whether SGI-4 can transfer from USDA15WA-1 to another bacterial strain, conjugation experiments were performed using a derivative (BBS 1356) of strain USDA15WA-1 marked with the *neo* gene (kanamycin resistance) in SGI-4 as a donor, and a nalidixic acid-resistant serovar Typhimurium strain (BSX 120) served as the recipient. Following plate mating experiments, transconjugates were obtained that were both kanamycin- and nalidixic acid-resistant, indicating SGI-4 transfer via conjugation. The conjugation frequency for transfer of SGI-4 from the BBS 1356 donor to the BSX 120 recipient was 8.88 × 10^−5^ per donor cell. An investigation by Branchu et al. [38] demonstrated conjugative transfer of SGI-4 when the donor and recipient were co-cultured in broth medium either in the presence of mitomycin C or under anaerobic conditions; an additive increase in conjugation frequency was observed in the presence of both stimulating conditions. Conjugation experiments by Arai et al. utilized a filter-mating method with a donor to recipient ratio of 1:9 and demonstrated SGI-4 transfer from serovar I 4,[5],12:i:- strain L-3841 to serovars Typhimurium, Heidelberg, Hadar, Newport, Cerro, and Thompson [39]. Our conjugation experiment was performed on LB agar medium with co-incubation of the donor and recipient strains at a 1:1 ratio with overnight incubation and, similar to Arai et al., demonstrates that extraneous induction of the conjugation factors is not a requirement for SGI-4 transfer.

Transconjugates from our conjugation experiments exhibited large and small colony morphologies. When streaked for single colony isolation onto LB agar, large colonies remained large, but frequently, isolation of small colonies produced a mixture of both small and large colony phenotypes. A similar occurrence was observed when small and large colonies were streaked from our frozen culture collection. To determine the genome location of the SGI-4 element in the transconjugates and ascertain whether differences in genome location explain the variation in colony morphology, genomic DNA sequencing and assembly of BSX 120 transconjugate strains were performed. Transconjugate BBS 1358^small^ contained a circular plasmid of SGI-4, whereas SGI-4 in BBS 1358^large^ and BBS 1359 had integrated into the chromosome at the *pheV* tRNA. Isolates BBS 1406 and BBS 1407 which were re-stocked as small and large colony morphology types from BBS 1358^small^, respectively, had SGI-4 replicating as a plasmid and integrated into the chromosome (*pheV*), respectively. DNA sequence analysis predicted that the multiplicity of the SGI-4 plasmid in strains BBS 1358^small^ and BBS 1406 was 2.63 and 2.36 copies per cell, respectively. In USDA15WA-1, SGI-4 is integrated into the chromosome at the *pheR* tRNA [6]. Arai et al. demonstrated chromosomal integration of SGI-4 at either *pheR* or *pheV* in transconjugates [39]. Therefore, DNA sequence analysis of transconjugates following SGI-4 transfer indicates that the MGE can replicate as a plasmid or integrate into either of the two phenylalanine tRNAs in the *Salmonella* chromosome. Additional information on plasmid content in BSX 120 transconjugates is available in Appendix B.

### 3.2. The Presence of SGI-4 in Strain USDA15WA-1 Enhanced Tolerance to Multiple Metals

**Figure 1:**
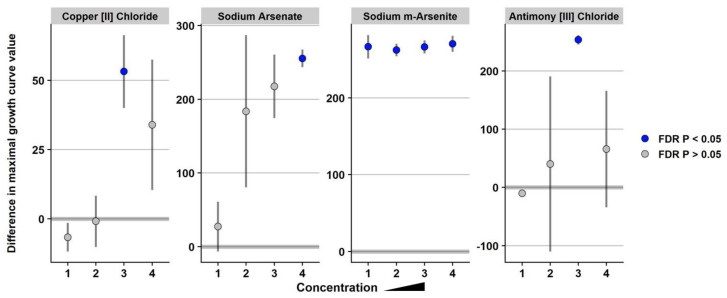
Significantly enhanced metal tolerance in *Salmonella* strains containing *Salmonella* Genomic Island 4. Analysis of *Salmonella* strains grown in Biolog Phenotype Microarrays identified significantly enhanced growth in copper chloride, sodium arsenate, sodium m-arsenite, and antimony chloride for strains USDA15WA-1 and BBS 1359 containing SGI-4 compared to strains BSX 120 and BBS 1270 lacking SGI-4. Strains were grown in 4 increasing concentrations of the chemical compounds in individual wells. Bacterial growth in wells with data points shown in blue is statistically significant (*p* < 0.05).

To determine whether the presence of SGI-4 containing multiple metal tolerance genes is advantageous (or detrimental) for bacterial growth in the presence of various chemical compounds, Biolog phenotype microarrays were performed using microplates 9–20. Strain BBS 1270 is a derivative of serovar I 4,[5],12:i:- isolate USDA15WA-1 whereby SGI-4 has been deleted, and strain BBS 1359 is a derivative of serovar Typhimurium strain BSX 120 that contains SGI-4 following conjugation with isolate BBS 1356 (Table 1). The absence of SGI-4 in strains BSX 120 and BBS 1270 did not significantly enhance tolerance to any of the chemical compounds on the Biolog phenotype microarray plates compared to *Salmonella* strains USDA15WA-1 and BBS 1359 containing SGI-4 (Table S2). In contrast, the presence of SGI-4 in *Salmonella* strains USDA15WA-1 and BBS 1359 significantly enhanced tolerance to sodium m-arsenite, antimony chloride, sodium arsenate, and copper chloride compared to strains BSX 120 and BBS 1270 that lack SGI-4 (Figure 1 and Table S2). An investigation by Branchu et al. [38] in the UK demonstrated that SGI-4 conferred a small but significantly enhanced tolerance to copper sulfate during aerobic and microaerobic growth for serovar I 4,[5],12:i:- strains; a significantly enhanced tolerance to copper sulfate due to SGI-4 in serovar I 4,[5],12:i:- strains was about 5-fold for anaerobically grown cultures compared to strains without SGI-4. Similarly, Arai et al. [39] in Japan demonstrated a 6-fold increase in the MIC of copper sulfate under anaerobic growth conditions for *Salmonella* isolates containing SGI-4 compared to strains without the metal island. Furthermore, the MIC of sodium arsenate was increased ≥128-fold for *Salmonella* strains containing SGI-4 grown aerobically compared to isolates lacking SGI-4. Our Biolog phenotype microarray data provide additional evidence that serovar I 4,[5],12:i:- isolates across the globe have enhanced tolerance to multiple metals due to the presence of SGI-4.

### 3.3. Copper Induced Gene Expression of Metal Tolerance Genes on Genomic Islands

**Figure 2:**
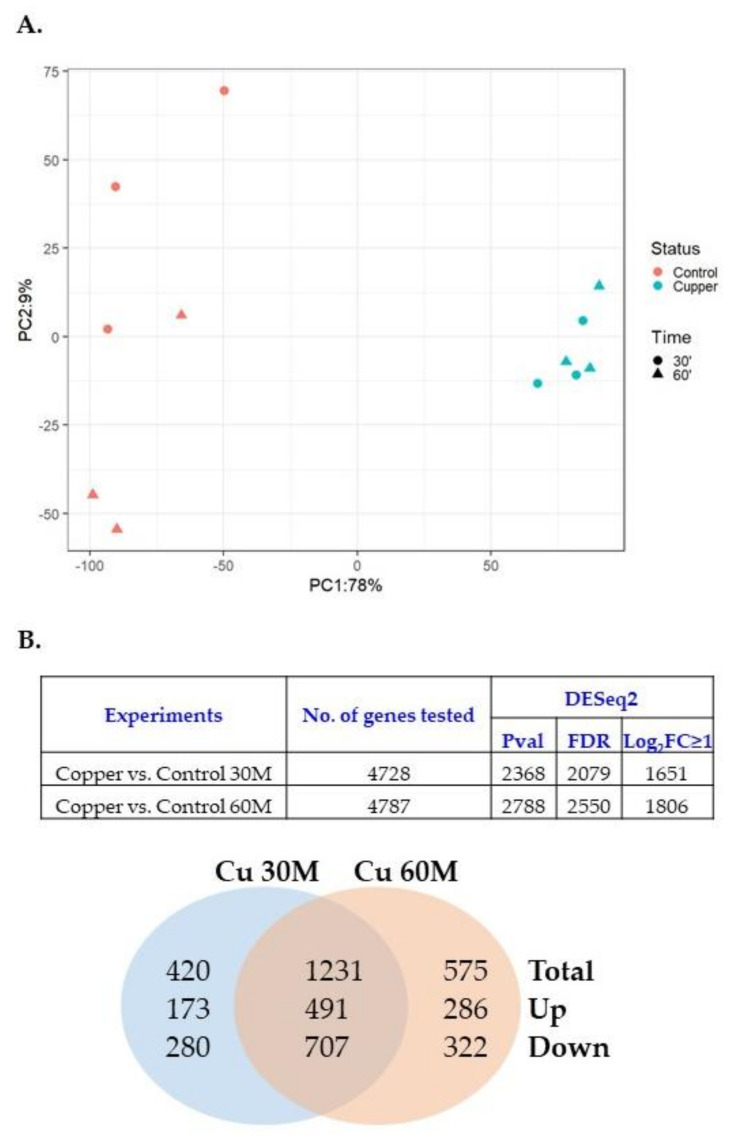
Differential expression of serovar I 4,[5],12:i:- genes following exposure to 5 mM copper sulfate. Strain SX 240 gene expression in the presence and absence of 5 mM CuSO_4_ after 30 and 60 min of exposure. Principal component analysis of the variation in gene expression +/− Cu exposure (**A**) and a Venn diagram of the number of unique or common genes differentially expressed (**B**).

Since the SGI-4 module contains copper tolerance genes, the transcriptional response of USDA15WA-1 to copper exposure was evaluated. Gene expression patterns were compared between USDA15WA-1 exposed to 0 and 5 mM CuSO_4_ for 30 and 60 min during the logarithmic growth phase using RNA-seq. Three biological replicates were generated for each condition (treated and control) and each timepoint (30 and 60 min). On average, 24.8 M reads per library (range from 21.7 to 31.4 M) aligned to the USDA15WA-1 genome (Table S3), and the reads cumulatively provided evidence for the expression of 4833 genes (a gene covered with at least one sequenced read in all RNA-seq libraries). Principal component analysis (PCA) revealed that separation of the no copper- (control) and 5 mM copper-exposed (30 and 60 min) libraries accounted for 78% variation in expression (Figure 2A). Compared to the no copper-exposed control samples, differential expression (|Log2FC ≥ 1|, FDR < 0.05) was observed for 1651 genes in response to 30 min of CuSO_4_ exposure (664 up-regulated and 987 down-regulated), and 1806 genes were differentially expressed following the 60-min exposure (777 up-regulated and 1029 down-regulated) (Figure 2B; Table S4 30 and 60 min tabs). Of the differentially expressed genes, 1231 were common between the 30- and 60-min copper exposure, and pathway analysis suggested a reorganization of metabolic processes, down-regulation of motility, and up-regulation of pathways involved in iron acquisition and utilization as well as the transport of and response to metal ions (Table S5). Although exposure was only to copper, gene expression induction was observed in metal tolerance genes located throughout the genome of USDA15WA-1 (Table 2). In the SGI-4 module, transcription of genes involved in arsenic, silver, and copper tolerance was significantly up-regulated. Copper genes located on the core genome were also transcriptionally induced. Furthermore, the expression of mercury tolerance genes located on the MDR module was up-regulated. Transcriptional induction of the mercury tolerance genes in response to metal exposure could possibly select for the maintenance of the MDR module containing the antimicrobial resistance genes.

Chalmers et al. [40] proposed a similar concept with the transfer of a *pco/sil* IncHI2 plasmid from *Escherichia coli* (*E. coli*) to *Salmonella* that resulted in elevated minimal inhibitory concentrations to copper. The investigators suggested that the enhanced copper tolerance gained by *Salmonella* may provide an advantage in copper-containing environments and, in turn, might co-select for antibiotic resistances on the plasmid. An investigation of 36 swine barns for heavy metal micronutrients in swine feed demonstrated a strong association between antimicrobial resistance and heavy metal tolerance in *Salmonella* serotypes important in public health [14].

Copper is an essential metal ion for bacteria, including *Salmonella*, but is toxic in excess [41]. A toxic effect of copper is the imbalance of Fe homeostasis and destabilization of iron-sulfur cluster proteins due to competition between Cu and Fe for binding sites [42]. To prevent improper metal substitution, expression of genes involved in Fe acquisition are often increased, just as we observed in our pathway analysis with up-regulation of genes involved in iron-sulfur clustering assembly, siderophore processes, and iron ion binding (Table S5). The greatest copper induction within the SGI-4 copper tolerance genes was observed for *pcoE* (Table 2). Zimmermann et al. described PcoE of *E. coli* as a periplasmic “metal sponge”, serving as a first line of defense against metal toxicity until the copper resistance operon *pcoABCD* (also present on SGI-4) is expressed [43].

Similar to the global response observed in our transcriptional profiling of serovar I 4,[5],12:i:- to copper (Table S5), Pontel et al. showed that, in addition to metal-specific regulatory networks, many global stress response pathways of serovar Typhimurium reacted to an excess of copper or zinc [44]. Lopez et al. linked genes involved in the copper stress response to the oxidative stress response in *Salmonella*, describing regulation of the *scsABCD* operon by the CpxR/CpxA signal transduction system [45]. CpxR/CpxA monitors bacterial envelope stress and regulates the ScsABCD system to prevent Cu toxicity and restore redox balance at the *Salmonella* envelope [46]. Our pathway analysis revealed up-regulation of several genes involved in cellular redox homeostasis and protein disulfide oxidoreductase activities, including the *scsCD* and *cpxA* genes (Table 2 and/or Table S5).

### 3.4. In-Feed Supplementation with Elevated Concentrations of Zn and Cu as an Antimicrobial Slowed the Decline of Salmonella I 4,[5],12:i:- Fecal Shedding in Pigs

With the elimination of sub-therapeutic antibiotic use for growth promotion, animal production industries are seeking alternatives to antibiotics and nutritional feed additives to benefit production performance. Inclusion of therapeutic/pharmacological concentrations of zinc (2000 to 4000 mg/kg) in feed has been reported to reduce diarrhea [47,48], enhance nursery pig performance via increasing average daily feed intake and average daily gain [49], improve innate and adaptive immunity [50], and reduce foodborne pathogens such as *Salmonella*, presumably by altering the composition of bacterial communities and metabolic properties [51], increasing intestinal villi height [52,53], and/or decreasing intestinal permeability to prevent translocation of pathogenic bacteria [54]. In addition to zinc, pharmacological/therapeutic concentrations of copper (100 to 250 mg/kg) have been reported to improve intestinal health and enhance growth performance, potentially due to bacteriostatic and bactericidal properties [55,56]. For example, Stahly et al. reported that dietary concentrations of copper sulfate in the range of 125–250 ppm optimized the performance of newly weaned piglets [57]. Furthermore, additive beneficial effects have been described for zinc and copper on swine growth performance [58,59].

**Figure 3:**
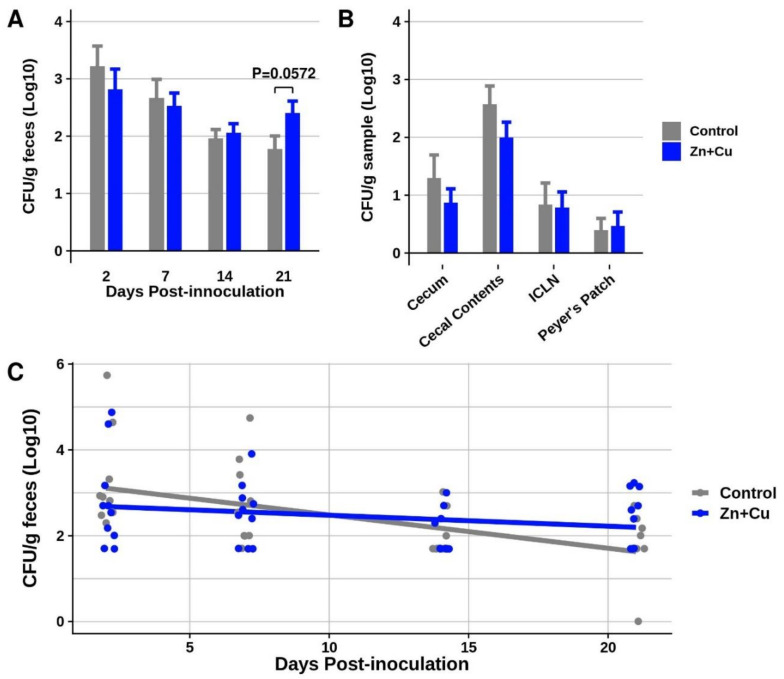
Swine fecal shedding and tissue colonization of serovar I 4,[5],12:i:- following inoculation in pigs with and without in-feed Zn+Cu supplementation. (**A**) Swine fecal shedding of SX 240 at days 2, 7, 14, and 21 following inoculation. (**B**) Swine tissue colonization of SX 240 at day 21 following inoculation. (**C**) Linear mixed model of swine fecal shedding of SX 240 over a 21-day period following inoculation.

In the current study, the in-feed effects of Zn+Cu (2000 mg/kg ZnO + 200 mg/kg CuSO_4_·5H_2_O) were evaluated for impact on shedding and intestinal tissue colonization of *Salmonella* I 4,[5],12:i:- in nursery-age pigs. Following four weeks of treatment (+/− Zn+Cu, *n* = 10 pigs/group), all pigs were inoculated with 8 × 10^7^ CFU of SX 240 and remained on their respective diets throughout the three-week study. In-feed Zn+Cu did not reduce the level of serovar I 4,[5],12:i:- fecal shedding over the 21-day study (Figure 3A), nor did Zn+Cu decrease intestinal tissue colonization (Figure 3B). In fact, at three weeks post-inoculation, the pigs receiving in-feed Zn+Cu tended (*p* = 0.0572) to shed higher levels of *Salmonella* in their feces. Using a linear mixed model to compare the trajectory of shedding over time, the model coefficients (Table S6) suggest that the shedding levels of *Salmonella* declined at a significantly (*p* = 0.020) slower rate over the three-week study in the pigs receiving in-feed Zn+Cu compared to the control pigs not receiving elevated Zn+Cu (i.e., more gradual slope of the blue line in Figure 3C). Potentially, the increased tolerance of serovar I 4,[5],12:i:- to metal exposure (Figure 1) due to the presence of metal tolerance genes on SGI-4 contributed to the persistence of *Salmonella* in pigs in the presence of in-feed Zn+Cu (i.e., selective pressure). Further investigation is required to determine the contributions of the metal tolerance genes on SGI-4 to swine colonization by serovar I 4,[5],12:i:- in the presence and absence of elevated metal(s) in the animal diet.

Other investigators have also suggested that the acquisition of metal tolerance genes is attributed to the use of metals (e.g., zinc and copper) as antimicrobial agents in human healthcare or in agriculture, and this practice has potentially contributed to the emergence of clinically relevant *Salmonella* isolates by aiding their survival in metal-contaminated settings, such as swine production [60,61]. Mourao et al. suggested that acquisition of the arsenic resistance gene *arsBII* by *Salmonella* I 4,[5],12:i:- has resulted in a foodborne pathogen resistant to arsenic compounds used in insecticides, herbicides, and coccidiostats [62]. Furthermore, a comparative whole-genome analysis of 50 epidemiologically unrelated serovar I 4,[5],12:i:- isolates from Italy revealed the widespread presence of heavy metal tolerance gene cassettes, with most of the *Salmonella* strains containing copper and silver resistance genes and half of the isolates also containing the mercury tolerance gene *merA* [7]. The authors propose that these genes might be useful for preventing the toxic effects of metals, thereby favoring the successful spread of *Salmonella* I 4,[5],12:i:- in farming environments.

### 3.5. Alterations in the Porcine Microbiota in Response to In-Feed Zn/Cu Treatment

**Figure 4:**
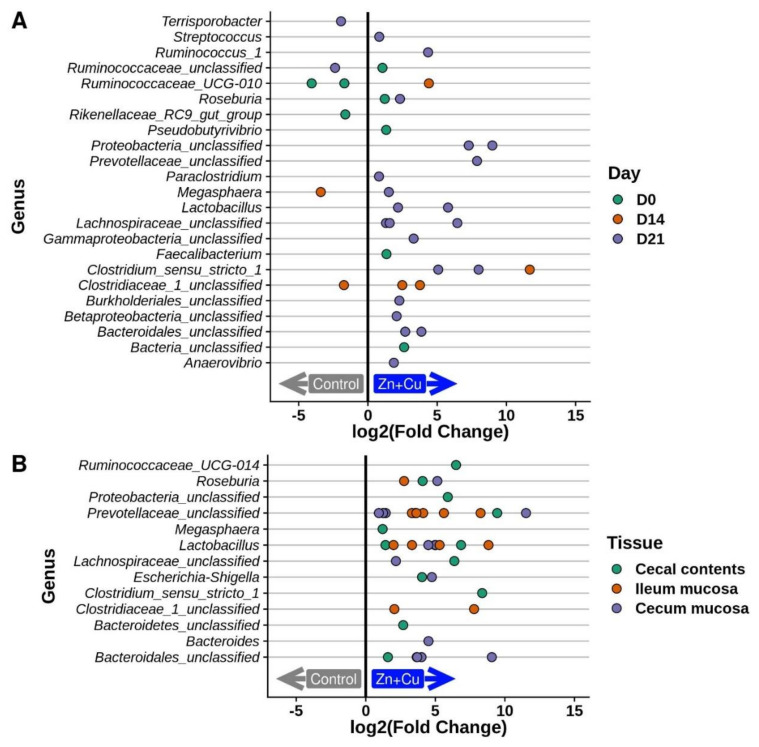
Differentially abundant operational taxonomic units (OTUs) from 16S rRNA gene-based fecal communities analysis. Tests compared control communities to Zn+Cu communities within each combination of timepoint (**A**) and tissue (**B**) independently. Each point represents one OTU, different OTUs from the same genus are displayed on the same line. Positive log2(FoldChange) value indicates enrichment in the “Zn+Cu” treatment group and a negative log2(FoldChange) value indicates enrichment in the “control” group. OTUs are classified according to the SILVA v132 taxonomy. Differential abundance was determined with DESeq2. Only OTUs with log2(FoldChange) > 0.75 and FDR *p* < 0.05 are shown.

To determine if in-feed Zn+Cu altered the swine gut microbiota, fecal microbial communities were compared between the two treatment groups (*n* = 10 pigs/group) at day 0 (following four weeks of Zn+Cu treatment but prior to serovar I 4,[5],12:i:- inoculation) and 2, 7, 14, and 21 d.p.i. At day 0, a near significant difference in the microbial communities between the +/− Zn+Cu groups was observed (*p* = 0.053, Table S7), and differentially abundant operational taxonomic units (OTU) were identified (Figure 4A,B). Once inoculated with *Salmonella*, no significant differences were observed in the microbial communities between the +/− Zn+Cu treatment groups at 2, 7, or 14 d.p.i. However, by 21 d.p.i., significant differences in bacterial community structure were observed between the two groups (Table S7). Several genera were identified as more abundant in the Zn+Cu-fed pigs compared to the control pigs in the feces at several timepoints (Figure 4A) as well as ileal mucosa, cecal mucosa, and cecal contents at 21 d.p.i. (Figure 4B). Although the significant differences in the microbial communities post-*Salmonella* inoculation coincided with greater serovar I 4,[5],12:i:- shedding levels in the pigs receiving in-feed Zn+Cu (i.e., 21 d.p.i.), it is unclear whether variations in the microbiome could be driving the *Salmonella* shedding differences, or if the more abundant levels of serovar I 4,[5],12:i:- in the pigs may be causing alterations in the microbiota.

It has been reported that the use of pharmacological concentrations of metals (such as zinc and copper) to improve growth performance of newly weaned piglets is partially attributed to the stability of the intestinal microbiota, particularly a large diversity of coliforms and a decrease in the presence of diarrhea-causing *E. coli* [63,64]. In our microbiota analysis, a greater abundance of several OTUs was observed in the feces and intestinal tissues of the pigs fed elevated concentrations of Zn+Cu, while only a few OTUs were enriched in the control group (Figure 4A,B). Several notable bacterial genera with OTUs enriched in the Zn+Cu pigs included the classic probiotic *Lactobacillus*, *Prevotella* which has been positively associated with luminal secretory IgA concentrations and body weight in pigs [65], and *Roseburia*. *Roseburia* spp. are Gram-positive, obligate anaerobic commensal bacteria that produce short chain fatty acids, especially butyrate, thereby potentially playing a role in maintaining gut health and immune defense [66]. Since a reduction in *Roseburia* spp. has been associated with diseases in humans such as irritable bowel syndrome, type II diabetes, and obesity, the organisms belonging to this genera have been suggested as biomarkers for intestinal pathologies as well as potential probiotics for the restoration of a health-associated gut microbiome [67]. In swine, *Roseburia* was classified as a core genus of the microbiome and members of this genus are producers of short chain fatty acids that contribute to swine health and development [68]. Even though elevated Zn+Cu administration in swine was associated with enrichment of health-associated genera (*Lactobacillus*, *Prevotella*, and *Roseburia*), the metal-containing diet may have also contributed to the colonization persistence of serovar I 4,[5],12:i:- containing SGI-4 encoding metal tolerance genes.

## 4. Conclusions

Our previous genomic analysis of USDA15WA-1, a *Salmonella* serovar I 4,[5],12:i:- isolate associated with a 2015 pork outbreak, revealed multiple antimicrobial resistance and metal tolerance genes encoded on two genomic insertions. In the current study, we demonstrated conjugative transfer of SGI-4 to a *Salmonella* recipient resulting in either plasmid replication or chromosomal integration in the *pheV* tRNA. Phenotypic analysis confirmed that serovar I 4,[5],12:i:- has increased tolerance to copper, arsenic, and antimony, and exposure to copper induced global transcriptional changes, including the induction of multiple metal tolerance genes (copper, arsenic, silver, and mercury). Administration of elevated levels of ZnO and CuSO_4_ in the swine diet did not reduce fecal shedding or tissue colonization and may have contributed to a slower decline in fecal shedding levels of serovar I 4,[5],12:i:- in the pigs (i.e., selective pressure). Altogether, the data corroborate current concerns in the literature that acquisition of metal tolerance genes may facilitate bacterial survival in diverse metal environments, potentially promoting the emergence and/or the expansion of clinically relevant *Salmonella* strains and possibly selecting for antibiotic resistance genes co-located on the genetic cassettes encoding the induced metal tolerance genes (i.e., co-selection of heavy metal-tolerant and multidrug-resistant *Salmonella*).

## Figures and Tables

**Table 1:** *Salmonella enterica* strains.

Strain No.	Strain Background	Genotype	Phenotype *	Source
BSX 137	USDA15WA-1, FSIS1503788, SX 238	serovar I 4,[5],12:i:-	ASSuT	Glenn Tillman FSIS [6]
SX 240	USDA15WA-1, FSIS1503788	serovar I 4,[5],12:i:-	ASSuT	Swine passaged [17]
BSX 120	UK-1, SB 26	serovar Typhimurium	Nal	[18]
BBS 1268	USDA15WA-1	pKD46-Gm	ASSuTGm, 30 °C	BSX 137/pKD46-GM; plasmid source [16]
BBS 1270	USDA15WA-1	ΔSGI-4: *neo*	ASSuTKn	BBS 1268/oBBI 528/529-*neo*
BBS 1356	USDA15WA-1	SGI-4Ω*neo*	ASSuTKn	BBS 1268/oBBI 542/543-*neo*
BBS 1358	UK-1	SGI-4Ω*neo*	NalKn	BSX 120 x BBS 1356
BBS 1359	UK-1	SGI-4Ω*neo*	NalKn	BSX 120 x BBS 1356
BBS 1406	UK-1	SGI-4Ω*neo*	NalKn	BBS 1358^small^
BBS 1407	UK-1	SGI-4Ω*neo*	NalKn	BBS 1358^large^

* Abbreviations for antimicrobial resistance are as follows: ampicillin (A), streptomycin (S), sulfisoxazole (Su), tetracycline (T), nalidixic acid (Nal), gentamicin (Gm), and kanamycin (Kn).

**Table 2:** Differential gene expression of serovar I 4,[5],12:i:- metal tolerance genes in response to copper exposure.

Gene ID	Gene Name	30 Min		60 Min		Description	Location
		Log_2_FC	FDR	Log_2_FC	FDR		
AOL22_00855	*cueO*	4.3	5.02 × 10^−34^	4.2	7.55 × 10^−31^	multicopper oxidase CueO	Core
AOL22_02225	*mdsC*	3	7.80 × 10^−9^	1.7	1.85 × 10^−9^	multidrug efflux transporter outer membrane subunit MdsC (GesC)	Core
AOL22_02230	*mdsB*	3.5	5.39 × 10^−8^	2.8	2.35× 10^−11^	multidrug efflux RND transporter permease subunit MdsB (GesB)	Core
AOL22_02235	*mdsA*	5.3	6.59 × 10^−12^	4.8	6.80 × 10^−16^	multidrug efflux RND transporter periplasmic adaptor subunit MdsA (GesA)	Core
AOL22_02240	*golT*	7	2.09 × 10^−28^	6.3	1.33 × 10^−18^	gold/copper-translocating P-type ATPase GolT	Core
AOL22_02245	*golS*	5.9	3.38 × 10^−29^	5	2.55 × 10^−27^	Au(I) sensor transcriptional regulator GolS	Core
AOL22_02250	*golB*	6.9	1.35 × 10^−97^	6.5	2.12 × 10^−102^	gold resistance metallochaperone GolB	Core
AOL22_02995	*copA*	5.7	2.64 × 10^−25^	5.2	4.89 × 10^−48^	copper-exporting P-type ATPase CopA	Core
AOL22_05910	*scsA*	2.4	9.70 × 10^−6^	4.1	1.13 × 10^−22^	copper resistance protein	Core
AOL22_05915	*scsB*	6.2	7.68 × 10^−37^	7.8	2.48 × 10^−88^	protein disulfide reductase	Core
AOL22_05920	*scsC*	5.4	4.80 × 10^−29^	6.7	1.42 × 10^−75^	disulfide bond formation protein DsbA	Core
AOL22_05925	*scsD*	4.8	4.60 × 10^−18^	6.4	2.60 × 10^−58^	protein disulfide oxidoreductase	Core
AOL22_14670	*merE*	1.8	2.10 × 10^−3^	1.3	6.18 × 10^−3^	broad-spectrum mercury transporter MerE	MDR
AOL22_14675	*merD*	1.4	7.46 × 10^−3^	1.5	1.72 × 10^−3^	mercury resistance co-regulator MerD	MDR
AOL22_14680	*merA*	1.3	1.40 × 10^−2^	1.1	1.09 × 10^−2^	mercury(II) reductase	MDR
AOL22_14685	*merC*	1.4	4.45 × 10^−3^	1.2	4.41 × 10^−3^	organomercurial transporter MerC	MDR
AOL22_14690	*merP*	1.4	3.21 × 10^−3^	1.1	5.62 × 10^−3^	mercury resistance system periplasmic binding protein MerP	MDR
AOL22_14695	*merT*	1.6	1.49 × 10^−3^	1.5	1.00 × 10^−3^	mercuric ion transporter MerT	MDR
AOL22_23325	*arsR*	2.6	1.35 × 10^−3^	2	1.65 × 10^−3^	transcriptional regulator	SGI-4
AOL22_23330	*arsD*	4.8	1.34 × 10^−9^	4	4.09 × 10^−7^	arsenite efflux transporter metallochaperone ArsD	SGI-4
AOL22_23335	*arsA*	4.6	4.25 × 10^−9^	3.7	1.39 × 10^−6^	arsenite efflux transporter ATPase subunit ArsA	SGI-4
AOL22_23340	*arsB*	4.5	9.60 × 10^−8^	3.9	1.56 × 10^−6^	arsenic transporter	SGI-4
AOL22_23345	*arsC*	3.7	2.37 × 10^−10^	3	1.23 × 10^−6^	arsenate reductase (glutaredoxin)	SGI-4
AOL22_23510	*silE*	9	2.29 × 10^−53^	7.5	1.55 × 10^−32^	silver-binding protein SilE	SGI-4
AOL22_23515	*silS*	2.7	4.93 × 10^−10^	2	1.42 × 10^−6^	copper/silver sensor histidine kinase SilS	SGI-4
AOL22_23520	*silR*	3.2	6.17 × 10^−8^	2.4	2.57 × 10^−6^	copper/silver response regulator transcription factor SilR	SGI-4
AOL22_23525	*silC*	7	1.04 × 10^−19^	6.5	6.81 × 10^−19^	Cu(+)/Ag(+) efflux RND transporter outer membrane channel SilC	SGI-4
AOL22_23530	*silF*	7.3	4.31 × 10^−32^	6.9	4.25 × 10^−23^	copper ABC transporter substrate-binding protein	SGI-4
AOL22_23535	*silB*	7.5	3.63 × 10^−25^	7	2.39 × 10^−21^	Cu(+)/Ag(+) efflux RND transporter periplasmic adaptor subunit SilB	SGI-4
AOL22_23540	*silA*	6.7	8.15 × 10^−26^	5.8	1.80 × 10^−19^	Cu(+)/Ag(+) efflux RND transporter permease subunit SilA	SGI-4
AOL22_23545	*orf105*	6.9	8.38 × 10^−204^	4.8	1.30 × 10^−64^	hypothetical protein	SGI-4
AOL22_23550	*silP*	5.7	1.94 × 10^−47^	3.5	1.66 × 10^−15^	Ag(+)-translocating P-type ATPase SilP	SGI-4
AOL22_23565	*pcoG*	2.3	6.85 × 10^−8^	0.71	5.20 × 10^−2^	copper resistance protein	SGI-4
AOL22_23570	*pcoA*	2.4	9.92 × 10^−19^	2.3	1.10 × 10^−25^	multicopper oxidase PcoA	SGI-4
AOL22_23575	*pcoB*	2.4	5.26 × 10^−16^	2.3	2.71 × 10^−17^	copper resistance outer membrane transporter PcoB	SGI-4
AOL22_23580	*pcoC*	2.5	4.30 × 10^−15^	2.1	2.41 × 10^−20^	copper resistance system metallochaperone PcoC	SGI-4
AOL22_23585	*pcoD*	1.7	4.43 × 10^−7^	1.4	4.73 × 10^−6^	copper resistance inner membrane protein PcoD	SGI-4
AOL22_23590	*pcoR*	0.4	2.43 × 10^−1^	0.7	2.27 × 10^−2^	copper response regulator transcription factor PcoR	SGI-4
AOL22_23595	*pcoS*	0.3	2.57 × 10^−1^	0.8	1.73 × 10^−4^	copper resistance membrane spanning protein PcoS	SGI-4
AOL22_23600	*pcoE*	7.7	6.64 × 10^−25^	6.7	1.68 × 10^−21^	copper-binding protein	SGI-4

## Supplementary Materials

The following are available online at https://www.mdpi.com/2073-4425/11/11/1291/s1. Table S1. PCR primers for recombineering; Table S2. Biolog Phenotype Microarray R pipeline analysis of USDA15WA-1, BSX 120, BBS 1270, and BBS 1359 with graphs of bacterial growth curves (blue) for significant metals; Table S3. Gene expression read counts; Table S4. SX 240 gene expression analysis following copper exposure; Table S5. Pathway analysis of differential gene expression of serovar I 4,[5],12:i:- following copper exposure; Table S6. Fixed effects from the linear mixed model describing swine fecal shedding of serovar I 4,[5],12:i:- over time; Table S7. Results of the PERMANOVA tests comparing microbial community structure between Zn+Cu and control groups; Table S8. SRA Biosample names for 16S rRNA gene amplicons for microbiota analysis.

## Appendix A

All raw data for genomic sequencing and RNA-seq are available through the SRA in Bioprojects PRJNA659233. Genomic sequencing data are available under accession numbers SAMN15950485–SAMN15950489. The raw RNA-seq reads are also available through the Gene Expression Omnibus (GEO) repository in GSE159054. RNA-seq data are available under accession numbers SAMN15950698–SAMN15950709 or GSM4818264–GSM4818275. Raw data for the 16s rRNA gene amplicon data are available through the SRA in Bioproject PRJNA638426 (see Table S8 for Biosample names).

## Appendix B

Strain USDA15WA-1 does not harbor a *Salmonella* virulence plasmid. Virulence plasmid pSTUK-100 present in the serovar Typhimurium recipient strain BSX 120 prior to conjugation (based on Nanopore GridIon sequencing and assembly of DNA extracted from the BSX 120 stock culture) was lost following conjugation with the BBS 1356 donor in transconjugate strains BBS 1358^small^ and BBS 1358^large^ that received SGI-4; loss of plasmid pSTUK-100 occurred regardless of whether SGI-4 was replicating as a plasmid or integrated into the chromosome. Sequencing data for BBS 1359 indicated that the ratio of the number of chromosome copies to pSTUK-100 was ~1/0.3, suggesting that only a subpopulation of transconjugate cells maintained the virulence plasmid, potentially due to unequal partitioning of pSTUK-100 during bacterial cell division in the broth culture grown for DNA extraction. Regions of the *Salmonella* virulence plasmid pStSR100 from serovar Typhimurium strain SR-11 were shown by Tinge and Curtiss [69] to hybridize to IncFII and IncFI plasmid incompatibility groups. Examination of the nucleotide sequence of pSTUK-100 from BSX 120 using PlasmidFinder-2.0 [70] also demonstrated the presence of plasmid incompatibility groups IncFIIs and IncFIBs. However, a plasmid incompatibility group was not identified in SGI-4 using PlasmidFinder-2.0, although SGI-4 contains genes encoding DNA partitioning functions. The frequency and consequences of pSTUK-100 loss in BSX 120 transconjugates warrants further investigation.
