# Supplementary material for: The Role of Salmonella Genomic Island 4 in Metal Tolerance of Salmonella enterica Serovar I 4,[5],12:i:- Pork Outbreak Isolate USDA15WA-1
Source: Genes (Basel). 2020 Oct 30;11(11):1291. doi: 10.3390/genes11111291 (PMC7716197; doi:10.3390/genes11111291)
Supplement: Supplementary file 1 [file genes-11-01291-s001.zip › genes-963783-suppl/Table S1 092320.pdf]

Table S1. PCR Primers for Recombineering.

Primer	Sequence*	Target
**oBBI 528**	5’ Phos-**GATGAGATCCTTAACGGGCGGAAAATGCAGGGTCCGGTCATACG**ATAGCTGAATGAGTGACGTGC	SGI-4 deletion
**oBBI 529**	5' **GCTACGGATTTCCATTCCCAGAAAAGCAAAAACCCAGCCATAGGC**ATAGAGCAGTGACGTAGTCGC	SGI-4 deletion
**oBBI 542**	5' **CACGATGGTGGGAACGATAAAAAAATTAATTCGGCAAACGGGGC**ATAGAGCAGTGACGTAGTCGC	SGI-4 insertion
**oBBI 543**	5' **GAGACTACGAACGATACCTGGACTGATAACGGGACCGCGACGCG**ATAGCTGAATGAGTGACGTGC	SGI-4 insertion

*Bold text indicates homology to the target gene and underlined text indicates homology to oBBI 92/93-*neo* for PCR amplification of the linear knockout fragment.
