# Supplementary material for: The Role of Salmonella Genomic Island 4 in Metal Tolerance of Salmonella enterica Serovar I 4,[5],12:i:- Pork Outbreak Isolate USDA15WA-1
Source: Genes (Basel). 2020 Oct 30;11(11):1291. doi: 10.3390/genes11111291 (PMC7716197; doi:10.3390/genes11111291)
Supplement: Supplementary file 1 [file genes-11-01291-s001.zip › genes-963783-suppl/Table S3 092320.pdf]

Table S3. Gene expression read counts.

Samples	Total No. reads sequenced	No. of reads aligned	No. of reads failed to align	Uniquely aligned reads	Multi-aligned reads
SX240_CON_M30_R1	28,585,903	23,398,788	5,187,115	13,779,926	9,618,862
SX240_CON_M30_R2	26,665,731	23,096,107	3,569,624	19,555,700	3,540,407
SX240_CON_M30_R3	26,928,218	22,240,032	4,688,186	21,934,979	305,053
SX240_CON_M60_R1	29,426,533	24,367,125	5,059,408	10,503,641	13,863,484
SX240_CON_M60_R2	26,446,499	22,745,698	3,700,801	22,540,464	205,234
SX240_CON_M60_R3	32,587,608	26,218,032	6,369,576	24,584,417	1,633,615
SX240_CUP_M30_R1	28,848,728	24,904,304	3,944,424	23,488,242	1,416,062
SX240_CUP_M30_R2	26,105,786	21,768,495	4,337,291	20,218,795	1,549,700
SX240_CUP_M30_R3	30,157,072	25,176,079	4,980,993	24,993,047	183,032
SX240_CUP_M60_R1	36,911,048	31,448,428	5,462,620	30,286,956	1,161,472
SX240_CUP_M60_R2	27,202,952	23,256,938	3,946,014	23,081,075	175,863
SX240_CUP_M60_R3	34,754,202	29,416,076	5,338,126	29,141,637	274,439
