# Supplementary material for: The Role of Salmonella Genomic Island 4 in Metal Tolerance of Salmonella enterica Serovar I 4,[5],12:i:- Pork Outbreak Isolate USDA15WA-1
Source: Genes (Basel). 2020 Oct 30;11(11):1291. doi: 10.3390/genes11111291 (PMC7716197; doi:10.3390/genes11111291)
Supplement: Supplementary file 1 [file genes-11-01291-s001.zip › genes-963783-suppl/Table S6 092320.pdf]

Table S6. Fixed effects from the linear mixed model describing swine fecal shedding of *Salmonella* over time.

Term	Estimate	std.error	Statistic	df	P value
(Intercept)	3.2625892	0.2467681	13.221276	52.05044	2e-16
day	-0.0777650	0.0154575	-5.030892	58.00002	5e-06
treatmentZn+Cu	-0.5307173	0.3489828	-1.520755	52.05044	0.134
day:treatmentZn+Cu	0.0522822	0.0218602	2.391662	58.00002	0.020

[#]The coefficients of the linear mixed model (table above) indicate that control pigs shed an average of 3.26 log(CFU) *Salmonella*/g feces at the beginning of the infection (intercept coefficient). While the coefficient for the Zn+Cu group intercept was negative (treatmentZn+Cu, coefficient=-0.53, p.value = 0.134), there is insufficient evidence to suggest that it significantly differed from that of the control group, suggesting that both groups had similar initial shedding values. The coefficient for 'day' (slope of the line for the control group) was -0.07 (p=5.02e-6), meaning that the shedding levels were estimated to decrease by 0.07 log(CFU) *Salmonella*/g feces with each day of the infection. The coefficient adjusting this slope for the Zn+Cu group (day:treatmentZn+Cu) was 0.052 and significantly different from zero (p=0.020), giving an estimate that the shedding values for the Zn+Cu decreased by only 0.025 log(CFU) *Salmonella*/g feces per day of the infection.
