# Supplementary material for: The Role of Salmonella Genomic Island 4 in Metal Tolerance of Salmonella enterica Serovar I 4,[5],12:i:- Pork Outbreak Isolate USDA15WA-1
Source: Genes (Basel). 2020 Oct 30;11(11):1291. doi: 10.3390/genes11111291 (PMC7716197; doi:10.3390/genes11111291)
Supplement: Supplementary file 1 [file genes-11-01291-s001.zip › genes-963783-suppl/Table S7 092320.pdf]

Table S7. Results of the PERMANOVA tests comparing microbial community structure between Zn+Cu and Control groups.

Comparison	F.Model	R2	P value	P FDR	Day	Sample
D0 feces Control vs D0 feces ZnCu	1.97	0.099	0.035	0.056	0	Feces
D2 feces Control vs D2 feces ZnCu	1.32	0.068	0.176	0.201	2	Feces
D7 feces Control vs D7 feces ZnCu	1.29	0.067	0.215	0.215	7	Feces
D14 feces Control vs D14 feces ZnCu	1.32	0.068	0.164	0.201	14	Feces
D21 feces Control vs D21 feces ZnCu	2.93	0.14	0.002	0.016	21	Feces
D21 ileum mucosa Control vs D21 ileum mucosa ZnCu	3.17	0.165	0.004	0.016	21	Ileum mucosa
D21 cecum mucosa Control vs D21 cecum mucosa ZnCu	2.56	0.124	0.02	0.04	21	Cecum mucosa
D21 cecal contents Control vs D21 cecal contents ZnCu	2.51	0.122	0.018	0.04	21	Cecal contents

The PERMANOVA tests (detailed above) suggest that the microbial communities in Zn+Cu fed pigs differed significantly from the microbial communities present in Control animals in all tissues at Day 21. In addition prior to Salmonella infection (Day 0) there was a nearly significant difference in the microbial communities between the two groups. PERMANOVA tests were conducted on Bray-Curtis dissimilarities which were derived from rarefied OTU tables. P values were corrected by the FDR method considering all tests that were performed.
